# Environmental sanitation: An ignored issue in India

**DOI:** 10.4103/0019-5278.40816

**Published:** 2008-04

**Authors:** Harshal T. Pandve

**Affiliations:** Department of Community Medicine, Dr. DY Patil Medical College, Pimpri, Pune, India

Dear sir,

In general, environmental sanitation covers arrangements for drainage of rainwater and effluents, collection and disposal of garbage and removal of human excreta. The World Health organization (WHO) defines environmental sanitation as “the control of all those factors in man′s physical environment, which exercise or may exercise a deleterious effect on his physical development, health and survival."[1] The Millennium Development Goals (MDG) is also insuring the environmental sustainability. Important indicators of the Millennium Development Goals are to reduce by half the proportion of people without sustainable access to safe drinking water and to achieve significant improvement in lives of at least 100 million slum dwellers, by 2020.[2]

India is still lagging far behind many countries in the field of environmental sanitation. Most cities and towns India are characterized by over-crowding, congestion, inadequate water supply and inadequate facilities of disposal of human excreta, wastewater and solid wastes.[3] Most of the problems in the country are due to defective environment, which in turn rob people of their health, destroy their livelihoods and undermine their overall development potential.[4] The environmental sanitation is still an ignored issue in India. To raise the overall of standard of living the issue of environmental sanitation must be tackled seriously. It is essential to take some stricter steps to raise the current state of environmental sanitation in India. For an example in Nigeria, the state government of Lagos has set up a sanitation court, which will prosecute offenders arrested for violating the state environmental sanitation exercise. The state is also planning to set up five additional mobile courts in this connection. Offenders could be any form of environmental pollution such as those concerning noise, water, air, littering or erecting posters and billboards without necessary approval.[5] Such steps taken by the governmental authorities certainly create an impact in the attitude of the community. In India, similar type of arrangements will be helpful in raising the current state of environmental sanitation.

To conclude, environmental sanitation involves both facilities provided by the governmental authorities as well as attitude of the community that work together to create a better environment.
